# Supplementary material for: Predicting nutrition and environmental factors associated with female reproductive disorders using a knowledge graph and random forests
Source: medRxiv. 2023 Jul 16:2023.07.14.23292679. Preprint. [Version 1] doi: 10.1101/2023.07.14.23292679 (PMC10371183; doi:10.1101/2023.07.14.23292679)
Supplement: Supplement 2 [file NIHPP2023.07.14.23292679v1-supplement-2.pdf]

medRxiv preprint doi: https://doi.org/10.1101/2023.07.14.23292679; this version posted July 16, 2023. The copyright holder for this preprint (which was not certified by peer review) is the author/funder, who has granted medRxiv a license to display the preprint in perpetuity.
It is made available under a CC-BY 4.0 International license .

SUPPLEMENTAL METHODS

OntoRunNER

OntoRunNER is a named entity recognition (NER) tool that reads strings of written content, which is then compared to a designated term list to identify what content is an exact or close match to the term list provided. For this project, we used OntoRunNER to coordinate strings of data input primarily regarding chemicals (medications or agricultural chemicals) to Chemical Entities of Biological Interest (ChEBI) Ontology terms. OntoRunNER allowed for both exact matches of the primary ChEBI label as well as synonyms of ChEBI content. It also allowed the string and ontology term to be considered a “match” if there were four or fewer different characters between them. Following use of OntoRunNER, we hand-reviewed terminology mappings for accuracy, which included additional manual mapping creation for common misspellings (e.g., ‘aderall’ versus ‘adderall’). We excluded from the dataset any string that could not be confidently mapped using OntoRunNER or manual means in an effort to avoid introducing inaccuracies.

Knowledge graph preparation for embedding

To prepare the graph for embedding using GRAPE, we removed all disconnected (singleton) nodes that shared no edges with other nodes as they provided little to no information for our link prediction model. We then selected the largest component of the graph, or the subgraph of the knowledge graph with the most nodes. Components were connected subgraphs of the primary graph, with the largest component the one with the highest number of nodes and thus offering the greatest capacity to develop link predictions. Using only the largest component removed 7.1% of respondents (n = 691) from the final analysis. As these respondents were not included in the largest component, it is likely the data available from their survey responses was insufficient for informing significant link predictions.

We used the DeepWalkSkipGram approach to learn the latent representations of the nodes within this graph network. This approach uses the Deep Walk deep learning approach, where nodes within the graph are treated like words, and random walks between nodes can be taken to create sentences [36]. The Skip-gram component includes inputting a single node and then contextualizing and classifying the word based on other words from the same sentence, allowing for projections of words coming both before and after the single word (node) of interest [42]. This approach to embedding allows for the creation of sentence structure using nodes and then the assessment of which nodes should be located near each other in the low-dimensional embedding visualization.

Logistic regression analysis

In more detail, the first two steps of supervised feature selection and random forest (RF) training were applied on 50 external stratified holdouts (train:test ratio 0.9:0.1). The variables that, on the average of all the holdouts, had an RF importance score greater than zero were chosen as the most important features.

For the first step of supervised feature selection on the training set, we ran preliminary experiments to choose among univariate feature selection techniques (where variables showing significant correlation with the label were selected), Boruta feature selection [49], permutation-based RF importance, and elastic nets (where the value of the elastic-net regularization

medRxiv preprint doi: https://doi.org/10.1101/2023.07.14.23292679; this version posted July 16, 2023. The copyright holder for this preprint (which was not certified by peer review) is the author/funder, who has granted medRxiv a license to display the preprint in perpetuity.
It is made available under a CC-BY 4.0 International license .

parameter λ is set via internal five-fold cross-validation and the α parameter balancing the amount of lasso and ridge constraints is set to 0.5) [50]. Given the comparable preliminary results, we opted for elastic nets due to their higher regularization capability, which results in a lower number of selected features. To avoid overfitting, we trained the RF by balancing the samples used to choose each split (sampsize) and chose the RF parameters (number of trees for the RF and the number "mtry" of variables considered to define each split) by a grid search on 100 internal rebalanced holdouts (train:test ratio = 0.9:0.1) to maximize the area under the precision-recall curve, which is appropriate in the case of imbalanced classes.

Next, for the directionality of scores, the important variables were used to train *logistic regression classifiers.* Considering how rare events resulting in highly imbalanced datasets may cause sharp logistic regression underestimates [48], we ran logistic regression on 100 holdouts rebalanced by undersampling. We averaged the results of the 100 iterations (odds and *P* values) to get the final estimates. We also calculated the variance inflation factor (VIF) and mean prevalence for each variable. These values are reported with the full logistic regression results in Supplemental Tables 2A-C. Of note, variables with a large VIF (>4) or a low mean prevalence score (0.001) may not be reliable regression outcomes due to colinearity or lack of sufficient data to determine the influence of a variable on the outcomes of interest.
